# Impact of COVID-19 on management and outcomes of NHS patients with recurrent respiratory papillomatosis: evidence from a UK registry

**DOI:** 10.1308/rcsann.2025.0030

**Published:** 2025-06-17

**Authors:** A Donne, K Keltie, P Cognigni, J Burn, S Powell, H Patrick, A Sims

**Affiliations:** ^1^ Alder Hey Children’s NHS Foundation Trust, UK; ^2^ The Newcastle upon Tyne Hospitals NHS Foundation Trust, UK; ^3^ University of Newcastle upon Tyne, UK; ^4^ National Institute for Health and Care Excellence, UK

**Keywords:** Human papillomavirus viruses, Pandemics, Health information systems, Treatment outcome, Patient safety

## Abstract

**Introduction:**

Patients with recurrent respiratory papillomatosis (RRP) require frequent surgical removal of benign growths in the airway to maintain patency. This study aimed to investigate the impact of the COVID-19 pandemic on these patients, by monitoring their care and outcomes before and after the pandemic.

**Methods:**

Participants were children or adults diagnosed with RRP, receiving treatment within an acute National Health Service hospital in the United Kingdom, registered with the Airway Intervention Registry. Data were captured between 1 April 2018 and 31 March 2022 (2 years pre- and post-COVID-19). Records for a subgroup of patients treated in England were linked to routine administrative data (Hospital Episode Statistics) for additional follow-up. Frequency of hospital visits, method of admission, type of surgical intervention, complications, disease severity (Derkay score) and voice quality were monitored.

**Results:**

There was a reduction in RRP surgery frequency post COVID-19. The reporting clinician noted an intervention delay caused by COVID-19 in 11.8% of cases, and in half of those the treating clinician noted that the delay had resulted in worse symptoms. Despite this, disease severity remained relatively stable in both children and adults, as demonstrated by the Derkay and voice quality scores.

**Conclusions:**

Patients with RRP experienced a reduction in surgical intervention post COVID-19. Although disease severity appeared overall stable within the study period, the long-term impact of changing surgical management of RRP patients in terms of voice quality and quality of life remain uncertain.

## Introduction

In March 2020, a COVID-19 lockdown occurred in the United Kingdom (UK) to limit the rate of spread and numbers of infected cases. Numerous medical organisations issued guidelines to advise on safe practice and NHS England initially advised a stop to routine surgery.^1^

It was expected that overall surgical activity would reduce in response to the COVID-19 guidelines.^2^ Published data on the operations for all surgical specialties recorded by surgical trainees in the UK dropped significantly between March 2020 and June 2021: recorded elective cases reduced by 53% and, perhaps surprisingly, emergency cases fell to 85% of the pre-March 2020 levels.^3^ Similar reductions in operation rates were reported in other European countries as a direct result of guidelines on control of elective surgery.^4^

One of the concerns driving this reduction was that COVID-19 represented a significant risk to healthcare workers. Because severe acute respiratory syndrome coronavirus 2 (SARS-CoV-2) is a respiratory virus, this risk was considered particularly relevant in the case of aerosol-generating procedures, and as a result could have been expected to particularly affect certain specialties, such as ear, nose and throat (ENT); indeed, in the United States, ENT surgery on children reduced by 50%.^5^ Interventions for maintaining a patent airway would not be considered routine; nevertheless, changes in practice brought on by the response to the COVID-19 pandemic may have affected these patients’ clinical management. We investigate the impact of the COVID-19 pandemic on patients requiring airway surgery using a national registry of recurrent respiratory papillomatosis (RRP) procedures that was established before the pandemic.

## Methods

### Data collections

Records from the Airway Intervention Registry: Recurrent Respiratory Papillomatosis data collection (AIR RRP) were extracted on 7 October 2022.^6^ The registry used a secure online database, only accessible through the National Health Service (NHS) network using password access. Unique registry passwords were given to end users who entered data. The users could only see information they had entered themselves. Each patient was designated a unique reference number within the database. Analysis was restricted to patients diagnosed with RRP attending an NHS hospital in England anytime between 1 April 2018 and 31 March 2022 inclusive to enable comparison of two full financial years before (2018/2019, 2019/2020) and after COVID-19 (2020/2021, 2021/2022). No restriction on date of RRP diagnosis was applied.

### Data linkage to routine administrative data

Owing to reduced access to research resources, data entry to the RRP registry was temporarily suspended during COVID-19 at some data collection centres. The registry was therefore linked to routine administrative data to determine hospital resource usage by RRP patients comprehensively across the NHS in England for linked patients. Pseudonymised data from the Hospital Episode Statistics (HES) and the Office of National Statistics mortality data set were requested through the Data Access Request Service and supplied by NHS Digital (DAR-NIC-17011-Z1B4J). Data linkage between the AIR RRP and HES was conducted for records across admitted patient care (APC) (including day cases), outpatients and mortality data sets between 1 April 2018 and 31 March 2022; linkage used age in years, sex, date of hospital visit and diagnosis codes. Patients for whom data linkage was unsuccessful and those with a recorded date of death preceding 31 March 2022 were excluded from analysis.

There is no specific diagnosis code in the International Classification of Diseases 10th revision (ICD-10) for RRP, therefore coverage of the RRP registry data collection (for England only) was estimated, based on the presence in HES records of the following: multiple admissions or day cases in the ENT specialty (defined using main specialty data field); discharge date between 1 April 2018 and 31 March 2022; containing generic diagnosis codes for either benign neoplasms of the airway (ICD-10 codes: D10, D14.1, D14.2, D14.3, D14.4) or human papilloma virus (ICD-10 code B97.7); requiring an airway intervention (any procedure code from the Office of Population Censuses and Surveys [OPCS]) classification of surgical operations chapter ‘E’).^7^ The number of laser procedures was determined from the procedure codes (OPCS codes E34.1: Microtherapeutic endoscopic extirpation of lesion of larynx using laser; E48.2: Fibreoptic endoscopic laser destruction of lesion of lower respiratory tract; E50.2: Endoscopic laser destruction of lesion of lower respiratory tract using rigid bronchoscope).

### Data cleaning

Where applicable, analysis was subgrouped by patient age as paediatric (<16 years) or adult. This categorisation was based on the age of the patient at their earliest visit recorded in the registry, and the patient remained in that subgroup throughout all analysis. From routine administrative data, outpatient visits were limited to those attended (including ‘seen’ and ‘late’), and those occurring within the ENT specialty.

### Outcome measures

Disease severity was monitored in the registry using Derkay score, which measures the extent of papillomatosis in specific anatomical areas.^6^ Voice quality was assessed through the GRBAS (grade, roughness, breathiness, asthenia, strain) scale, Voice Handicap Index (VHI) and paediatric VHI. Additional details including frequency of hospital visits, type of intervention (subtype of surgery including micro-debrider, laser, cold steel, radiofrequency ablation and use of adjuvant therapies), and surgical procedure duration were also obtained from the registry.

From the routine administrative data, the number of admissions, admission method (elective or urgent), length of stay, proportion of day cases and number of outpatient attendances were identified. Reflective of clinical practice, frequency of surgery was considered as a surrogate marker of disease severity. Respiratory procedures were identified in the APC data set of the HES (which includes day cases) through procedure codes (OPCS codes within the ‘E’ chapter appearing in any OPRTN 01-24 data fields). In-hospital complications were identified from clinical coding practice of diagnosis codes.^8^ Laboratory-confirmed diagnosis of COVID-19 was identified through diagnosis code (ICD-10) U07.1.

Outcomes were reported for paediatric and adult subgroups, with financial year (2018/2019, 2019/2020, 2020/2021, 2021/2022) acting as a proxy in defining pre-COVID-19 (on or before 31 March 2020) and post-COVID-19 (after 31 March 2020) periods.

Additional data fields were added to the AIR RRP registry on 20 July 2020 to capture information about delays in care due to COVID-19. Responses to these additional COVID-19 data fields were summarised in descriptive statistics due to their non-comparative nature.

### Ethical considerations

The Airway Intervention Registry gained favourable ethical opinion as a research database on 29 December 2014 (IRAS for original application: 164160, renewal: 288078). Patient/parent consent was required before data were permitted in the online database.

### Statistical analysis

All scripts for applying eligibility criteria, data cleaning, processing and statistical analysis were written in the statistical programming language R.^9^

## Results

**Figure 1:**
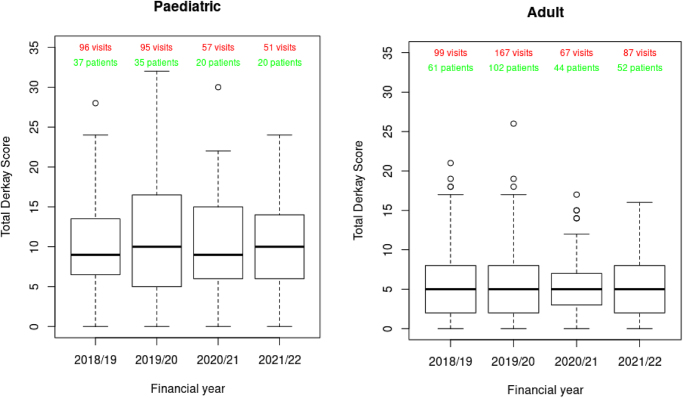
Total Derkay scores recorded in the Airway Intervention Registry: Recurrent Respiratory Papillomatosis, reported separately for children and adult patients over time. Note: visits without a Derkay score were excluded from this figure.

As of 7 October 2022, a total of 330 patients and 1,603 hospital visits were recorded in the database (Appendix 1 – available online). Of these, 284 patients attended an NHS hospital in England within the study period. A total of 365 visits that took place before 1 April 2018 and 63 visits that took place after 31 March 2022 were excluded. A total of 241 patients (189 adults, 52 children) with 1,044 hospital visits between 1 April 2018 and 31 March 2022 inclusive (including admissions, day cases, outpatient visits and emergency department attendances) from the registry remained for analysis (Table 1). This comprised 648 visits in 198 patients prior to COVID-19 (1 April 2020) and 396 visits in 132 patients after COVID-19 (Appendix 1 – available online). The median Derkay score (across visits where it was reported), remained stable between 2017 and 2022 (Figure 1) and was significantly higher in children (indicating more severe disease) than in adults. Trends in voice quality were difficult to determine from small numbers (GRBAS, pVHI and VHI are given in Appendix 1 – available online).

**Table 1:** Demographics of included patients (from registry) at baseline as recorded in the AIR RRP registry

	Paediatric	Adult
Participants	52	189
Age at diagnosis, years	3 [2:6]	35 [26:47]
Male sex, *n* (%)	33 (63.5%)	126 (66.7%)
Ethnicity	*n *= 52	*n *= 174
White	45 (86.5%)	163 (93.7%)
Asian/Asian British	2 (3.8%)	3 (1.7%)
Black/African/Caribbean/Black British	1 (1.9%)	4 (2.3%)
Mixed/multiple ethnic origin	4 (7.7%)	1 (0.6%)
Other	0 (0%)	3 (1.7%)
Derkay	9 [7:12] (*n *= 46)	6 [4:9] (*n *= 138)
pVHI	44 [24:56] (*n *= 13)	–
VHI	–	59 [33:73] (*n *= 72)
GRBAS	7 [4.8:10.5] (*n *= 28)	4 [2:8] (*n *= 88)

Age, Derkay and voice assessment scores are shown as median [Q1:Q3]

AIR RRP = Airway Intervention Registry: Recurrent Respiratory Papillomatosis data collection; GRBAS = grade, roughness, breathiness, asthenia, strain; pVHI = paediatric Voice Handicap Index; VHI: Voice Handicap Index

Clinicians recorded that patient treatment was delayed in 11.8% of visits post-COVID-19 (44 of 374 visits in 29 patients): 34 because of hospital delay, 3 were delayed because the patient had COVID-19 and 7 for other reasons (including suspected COVID-19 in patient or parent). No delays were due to the patient or family member isolating, or the patient or parent choosing to decline hospital attendance because of COVID-19 concerns. These delays had clinical significance: in 52% (23 of 44 visits in 21 patients) the treating clinician noted that the delay had resulted in worse patient symptoms, in their opinion, including hoarseness or quality of breathing. In 5.1% (7 of 137) of visits post-COVID-19, a documented change was made to the intended anaesthetic airway (e.g. tubed anaesthesia), only one visit recorded using a different surgical technique than previously planned, and no visits recorded additional medical management. Of the 199 visits that recorded additional measures taken because of COVID-19, 123 reported use of personal protective equipment (PPE), 25 exhaust system, 23 powered hood, and 14 double gloving.

**Figure 2:**
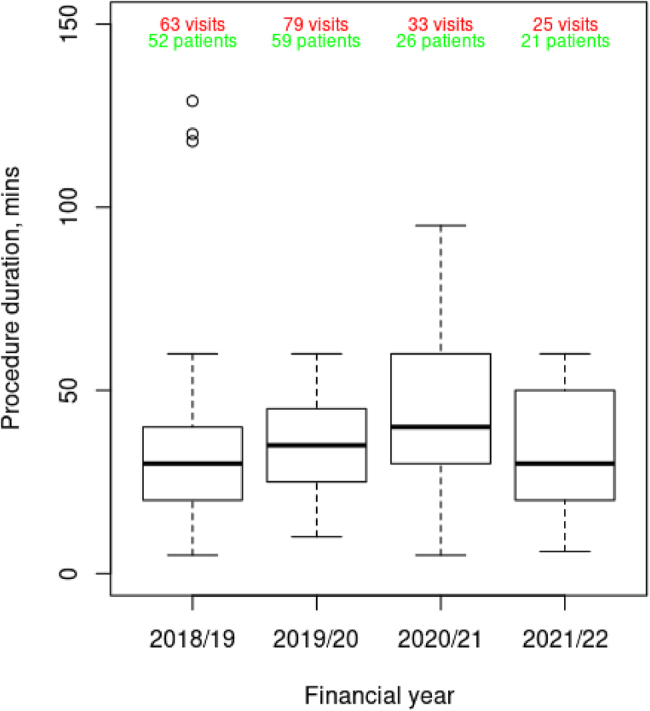
Recurrent respiratory papillomatosis surgery duration (min) recorded in the Airway Intervention Registry: Recurrent Respiratory Papillomatosis by fiscal year.

The median procedure duration increased in 2020/2021, potentially related to increased surgeon PPE and decontamination procedures, but was also more variable (increased interquartile range) (Figure 2). In 2020/2021, a reduction in laser use corresponded with an increase in cold steel surgical management; however, use of adjuvant therapies has remained relatively stable (Appendix 1 – available online).

### Hospital Episode Statistics

According to HES APC data (which includes day cases), 410 patients in England had more than two ENT admissions or day cases with a diagnosis suggestive of RRP and a respiratory intervention during the study period. This would suggest that the RRP registry captured 59% of RRP cases in England (241 of 410).

**Figure 3:**
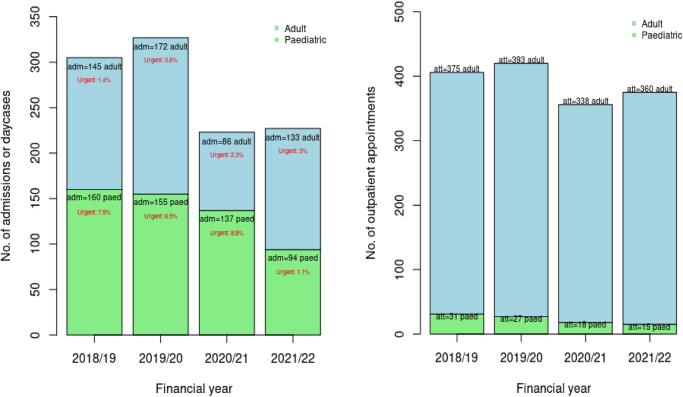
Number of hospital visits from Health Episode Statistics, including (a) admissions and day cases (adm) within the admitted patient care data set containing a respiratory procedure for matched patients; 155 adults and 50 children; and (b) ear, nose and throat outpatient attendances (att) for matched patients; 167 adults and 17 children for matched patients, over time.

A total of 220 patients (91%; 169 adults, 51 children) from the registry were successfully data linked to HES. This included 1,082 inpatient all-cause admissions or day cases involving respiratory interventions (surgical interventions, diagnostic imaging), with 191 including a laser procedure (17.7% in 2018/2019, 19.9% in 2019/2020, 13.0% in 2020/2021 and 20.3% in 2021/2022). Two cases contained a laboratory-confirmed diagnosis of COVID-19. There was a reduction in admissions (day case and inpatient) for the linked patients in 2020/2021 in both adult and paediatric RRP patients, which corresponds to the first year of the pandemic. Activity in adults in 2021/2022 is similar to activity pre-COVID-19; however, this has not yet recovered in paediatric cases (Figure 3). The majority of admissions were day cases (steadily increasing in children; Appendix 1 – available online), but in-hospital complications were rare (Appendix 1 – available online), with no patient requiring an admission to an intensive care unit. Urgent admissions appear variable, making it difficult to identify trends (Figure 3a) indicating that RRP patients should continue to be monitored regularly.

Data linkage to the HES also identified 1,557 outpatient attendances within ENT (1,466 adult and 91 paediatric). A reduction in outpatient attendances was observed corresponding with the first year of the pandemic; however, activity in 2021/2022 was similar to that pre-COVID-19 in adults (Figure 3b). Recovery to pre-COVID-19 hospital attendance levels has not yet been achieved in paediatric RRP cases.

## Discussion

The AIR RRP data collection was the only registry within ENT that was active before and during COVID, making it best placed to monitor hospital resources over time. This study showed a reduction in the hospital activity post-COVID-19 in both adults and children. The procedural activity around RRP in adults did increase again in 2021, but remained low in children. This may indicate a change in approach to the management of RRP in general as a result of the COVID-19 pandemic. Following national guidance, many clinics changed to virtual clinics (telephone or video), which are subjectively easier to attend.^10^ The total number of outpatient clinics each year did not change significantly during the study period.

Hospital visits for surgical management of RRP were further complicated by PPE and ventilation issues, which introduced a new way of operating. The burden of PPE use for otolaryngology (airway procedures) was significant because of the use of highly restrictive FFP3 or a powered air purifying respirator.^11^ The latter was particularly difficult to operate in and this affected communication in theatre.^12^ Accordingly, we observed longer surgeries during 2020 and the interquartile range also increased. This suggests that, in some cases, pandemic-related restrictions (e.g. PPE requirements and increased cleaning procedures) had a significant impact on the time of surgery, a finding reflected in other surgical specialties’ experiences.^12^ On the other hand, the proportion reporting the use of PPE was only 123 of 199 visits – which is surprising. This might indicate inadequate data entry recorded in retrospect (the data entry person not being the primary surgeon) or perhaps a genuine lack of PPE due to supply shortages. Because concerns around aerosol-generating procedures were initially high, surgeons might have adopted different operative techniques to accommodate the transmission risk. Powered instruments and suction were initially thought to be high risk for generating an aerosol, which could explain the reduction in use of micro-debrider found in our study; however, this is also the predominant method for papilloma control in children, and there were fewer children in our study. Adjuvant therapy remained at a stable rate throughout this 4-year study period, suggesting that patients were not progressing in severity, and this is confirmed by the relatively constant median Derkay scores reported; however, the authors acknowledge that multiple Derkay scores were reported in some patients and none in others, which may bias these results. A stable Derkay score over time could also be explained by a natural slowing or plateau in the anatomical extent of growth of papillomas, which may occur at a different point for each patient. There may have been reasons not to introduce adjuvant therapies during COVID-19 because this may have resulted in increased exposure due to an increased number of visits for the adjuvant to be administered. Furthermore, the registry did not record anxiety and depression scores, which are known to be influenced by long waiting lists.^13^ In addition, there was undoubtedly increased stress and anxiety for medical and nursing professionals during the COVID-19 period. However, voice quality scores (as assessed by clinician and patient reported) were variable during the study period, and impacted by small numbers. Furthermore, the study team acknowledge that data entry into the registry was temporarily suspended in some centres during COVID-19 because of a lack of staff resources. This may have influenced the results because different surgeons may have performed surgery on behalf of absent colleagues and their own techniques may have varied or they may have had to change to accommodate aerosol safer practice. It would seem unlikely that a surgeon new to a case would change the technique used by the regular surgeon in usual circumstances. This is perhaps indicated by the adjuvant therapy rate remaining stable. The extent of disease removal may have changed either more or less, but this would be difficult to prove with this data set. Other biases might have been that surgeons adapted to actively prolong intervals between surgeries.

Our data show that airway surgery (which included diagnostic imaging and therapeutic procedures and therefore provided an overestimate) did still occur during COVID-19, albeit to a lesser extent in children than in adults. This appeared to be sufficient for clinical management, because Derkay scores remained similar.

### Study limitations

This study linked a clinical registry to a routine database (HES) to gain additional longitudinal follow-up information on RRP patients being treated in the NHS; however, the limitations of using routinely collected data remain. There is no specific diagnosis code (ICD-10) for RRP, therefore routinely collected data may include other diseases that require repeated respiratory intervention, which could lead to an underestimation of coverage of the registry. In addition, procedure type cannot be fully differentiated from existing procedure (OPCS4.9) codes recorded in HES; however, the number of surgical ENT visits and proportion using lasers in HES followed a similar trend to that seen in the registry. Increased activity in other forms of ENT surgery have been documented post-COVID-19; for example, temporary tracheostomy rates (using E42.3 OPCE 4.9 code) increased from 8,618 in 2019/2020 to 12,227 in 2020/2021.^14,15^ This is, however, confounded by the possibility that tracheostomy was performed as a direct result of prolonged intubation for COVID-19. This study also contains a small number of patients with variable duration of RRP disease; however, it remains the largest and richest data set of RRP patients within the UK.

Frequency of surgery was considered as a surrogate marker of disease severity and not an outcome measure because this may be influenced by other factors such as local policy, theatre or staff availability. If patients had consciously decided not to present for a variety of reasons during the COVID-19 pandemic or if their condition had progressed significantly, they would be expected to return as emergencies. The urgency rates of admission are small across the 4-year period 2018–2021, although it is encouraging to see that the rate for paediatric cases in the 12 months between 2021 and 2022 was 0%, suggesting that the monitoring was correct. We suspect that increased use of virtual clinics allowed safe monitoring. This might indicate that parameters around RRP patients’ need for surgery may benefit from a review to avoid overtreatment.

## Conclusion

The COVID-19 pandemic has affected the provision of healthcare to a dramatic degree, and perhaps more so in those specialties in which aerosol-generating procedures are common, like ENT. When looking at regular treatment of the chronic airway condition RRP, there was a reduction in hospital activity during 2020. In adults, the 2020 reduction in respiratory procedures was followed by a return to pre-pandemic levels the following year, but this was not the case in paediatric respiratory procedures or in paediatric clinic attendances. In addition, it is possible that hospitals were more actively adopting a patient-initiated follow-up approach during COVID, leading to longer intersurgical intervals, which helped lighten the theatre burden during the COVID-19 pandemic when beds were otherwise full. It is unclear whether the change in hospital activity for children with RRP is a consequence of a change in surgical management, or a consequence of natural disease progression. In the short term (2 years) covered by this study, the indications are promising that this change in surgical management has not had a negative impact, because neither Derkay scores nor emergency attendances have increased. However, the long-term impact of the observed change in RRP management, including prolonged periods between surgery, on voice quality and quality of life will only become apparent in the years to come. If the current findings are replicated over longer periods, it may suggest that a less aggressive, more patient-initiated surgical maintenance schedule may be equally effective for children with RRP.

## Conflicts of interest

HP is employed by the National Institute for Health and Care Excellence (NICE). KK, PC, JB and AJS are employed by the Newcastle upon Tyne Hospitals NHS Foundation Trust, which hosts the Newcastle External Assessment Group and received funding from NICE. The views expressed in this publication are those of the authors and not necessarily those of NICE.

## Funding

This research was funded by the National Institute for Health Research (NIHR) under its Research for Patient Benefit (RfPB) Programme (grant reference number PB-PG-0416-20037) and NICE (reference: RX261, call-off order number NICE1511). The views expressed are those of the author(s) and not necessarily those of the NIHR or the Department of Health and Social Care. The research team acknowledges the support of the National Institute for Health Research Clinical Research Network (NIHR CRN).

## Author contributions

All authors participated in the study conception and design. The Young Persons Advisory Group North of England (YPAGne) contributed to the study design, recruitment approach, and publicity of results. Newcastle Advising Patient Experience (APEX) and NIHR VOICE patient groups contributed to recruitment approach and publicity of results. KK, PC and JB extracted and analysed the data. All authors participated in the interpretation of the data, drafted the article, or revisited it critically for important intellectual content. AJS had full access to all of the data in the study and is the study guarantor.

## Data availability statement

Any external researchers requesting data from the Airway Intervention Registry will be required to submit a formal application form. Only formal applications with appropriate ethical approvals in place (reviewed by an independent ethics committee) will be reviewed by the established AIR Steering Group committee. If approval is given, only anonymised data will be shared for the purposes of external research studies.

## References

[1] NHS England. Next steps on NHS response to COVID-19. https://www.england.nhs.uk/coronavirus/wp-content/uploads/sites/52/2020/03/urgent-next-steps-on-nhs-response-to-covid-19-letter-simon-stevens.pdf (cited March 2023).

[2] Mahendran GN, Tey CS, Musso MF *et al.* Measuring the impact of a delay in care on pediatric otolaryngologic surgery completion. *Ear Nose Throat J* 2022: 1455613221134428. 10.1177/01455613221134428 36240145

[3] Lund J, Sadler P, McLarty E. The effect of COVID-19 on surgical training. *Surgery (Oxf)* 2021; **39**: 829–833. 34545261 10.1016/j.mpsur.2021.09.003 PMC8443340

[4] Riemann S, Speck I, Gerstacker K *et al.* Collateral damage of the COVID-19 pandemic: an alarming decline in critical procedures in otorhinolaryngology in a German university hospital. *Eur Arch Otorhinolaryngol* 2021; **278**: 3417–3423. 33320294 10.1007/s00405-020-06519-1 PMC7736669

[5] Tran K, Cimon K, Severn M *et al.* Aerosol generating procedures and risk of transmission of acute respiratory infections to healthcare workers: a systematic review. *PLoS ONE* 2012; **7**: e35797. 22563403 10.1371/journal.pone.0035797 PMC3338532

[6] Sims A, Keltie K, Belilios E *et al.* Our experience in developing and operating the airway intervention registry for recurrent respiratory papillomatosis (AIR-RRP): national data collection [version 2; peer review: 2 approved]. *NIHR Open Res* 2023; **2**: 22. 36855411 10.3310/nihropenres.13244.2 PMC7614251

[7] Donne AJ, Keltie K, Cole H *et al.* Prevalence and management of recurrent respiratory papillomatosis (RRP) in the UK: cross-sectional study. *Clin Otolaryngol* 2017; **42**: 86–91. 27208548 10.1111/coa.12683

[8] Aylin P, Tanna S, Bottle A, Jarman B. How often are adverse events reported in English hospital statistics? *BMJ* 2004; **329**: 369. 15310606 10.1136/bmj.329.7462.369 PMC509338

[9] R Core Team. *R: A Language and Environment for Statistical Computing*. Vienna, Austria: R Foundation for Statistical Computing. http://www.R-project.org/ (cited March 2023).

[10] National Institute for Health and Care Excellence. *NICE publishes new COVID-19 rapid guideline on arranging planned care in hospitals and diagnostic services*. https://www.nice.org.uk/news/article/nice-publishes-new-covid-19-rapid-guideline-on-arranging-planned-care-in-hospitals-and-diagnostic-services (cited March 2023). 33497146

[11] Frauenfelder C, Butler C, Hartley B *et al.* Practical insights for paediatric otolaryngology surgical cases and performing microlaryngobronchoscopy during the COVID-19 pandemic. *Int J Pediatr Otorhinolaryngol* 2020; **134**: 110030. 32278168 10.1016/j.ijporl.2020.110030 PMC7142686

[12] Karachalios T, Maasalu K, Felländer-Tsai L. Personal protection equipment for orthopaedic and trauma surgery during the COVID-19 pandemic: the results of an EFORT survey initiative. *EFORT Open Rev* 2022; **7**: 122–128. 35192510 10.1530/EOR-21-0120 PMC8897563

[13] Gagliardi AR, Yip CYY, Irish J *et al.* The psychological burden of waiting for procedures and patient-centred strategies that could support the mental health of wait-listed patients and caregivers during the COVID-19 pandemic: a scoping review. *Health Expect* 2021; **24**: 978–990. 33769657 10.1111/hex.13241 PMC8235883

[14] NHS Digital. *Hospital admitted patient care activity, 2019–20: Procedures and interventions*. https://files.digital.nhs.uk/20/0864E6/hosp-epis-stat-admi-proc-2019-20-tab.xlsx (cited July 2023).

[15] NHS Digital. *Hospital admitted patient care activity, 2020–21: procedures and interventions*. https://files.digital.nhs.uk/A6/43CDC1/hosp-epis-stat-admi-proc-2020-21-tab.xlsx (cited July 2023).

